# Development of START-EDI guidelines for reporting equality, diversity and inclusion in research: a study protocol

**DOI:** 10.1136/bmjopen-2024-095778

**Published:** 2025-07-16

**Authors:** Michael G Fadel, Hannah Kettley-Linsell, Piers R Boshier, Rebecca Barnes, Christopher Newby, Anthony Muchai Manyara, Peter Buckle, Darshali A Vyas, Julie Hepburn, Philip Edgar-Jones, Tanvi Rai, Brian D Nicholson, Amanda J Cross, Linda D Sharples, Sally Hopewell, Jérémie F Cohen, Vivian Welch, Patrick MM Bossuyt, George B Hanna

**Affiliations:** 1 Department of Surgery and Cancer, Imperial College London, London, UK; 2 NIHR HealthTech Research Centre in In Vitro Diagnostics, London, UK; 3 Department of Population Health Sciences, University of Leicester, Leicester, UK; 4 NIHR Research Support Service Hub, University of Leicester and Partners, Leicester, UK; 5 Medical School, University of Nottingham, Nottingham, UK; 6 Global Health and Ageing Research Unit, Bristol Medical School, University of Bristol, Bristol, UK; 7 Department of Medicine, Division of General Medicine, Harvard Medical School and Massachusetts General Hospital, Boston, Massachusetts, USA; 8 Public Involvement Community, Health and Care Research Wales Support Centre, Cardiff, UK; 9 Sky Arts and Entertainment, London, UK; 10 Nuffield Department of Primary Care Health Sciences, University of Oxford, Oxford, UK; 11 Department of Medical Statistics, London School of Hygiene and Tropical Medicine, London, UK; 12 Oxford Clinical Trials Research Unit / Centre for Statistics in Medicine, Nuffield Department of Orthopaedics, Rheumatology and Musculoskeletal Sciences, University of Oxford, Oxford, UK; 13 Centre of Research in Epidemiology and Statistics (CRESS), INSERM, OPPaLE Research Team, Université Paris Cité, Paris, France; 14 Department of General Pediatrics and Pediatric Infectious Diseases, Necker-Enfants Malades Hospital, Assistance Publique-Hôpitaux de Paris, Université Paris Cité, Paris, France; 15 Methods Centre, Bruyère Research Institute, Ottawa, Ontario, Canada; 16 Campbell Collaboration, Philadelphia, Pennsylvania, USA; 17 Department of Epidemiology and Data Science, Amsterdam University Medical Center, Universiteit van Amsterdam, Amsterdam, The Netherlands

**Keywords:** Health Equity, Delphi Technique, Protocols & guidelines

## Abstract

**Abstract:**

**Introduction:**

Acknowledging equality, diversity and inclusion (EDI) in research is not only a moral imperative but also an important step in avoiding bias and ensuring generalisability of results. This protocol describes the development of STAndards for ReporTing EDI (START-EDI) in research, which will provide a set of minimum standards to help researchers improve their consistency, completeness and transparency in EDI reporting. We anticipate that these guidelines will benefit authors, reviewers, editors, funding organisations, healthcare providers, patients and the public.

**Methods and analysis:**

To create START-EDI reporting guidelines, the following five stages are proposed: (i) establish a diverse, multidisciplinary Steering Committee that will lead and coordinate guideline development; (ii) a systematic review to identify the essential principles and methodological approaches for EDI to generate preliminary checklist items; (iii) conduct an international Delphi process to reach a consensus on the checklist items; (iv) finalise the reporting guidelines and create a separate explanation and elaboration document; and (v) broad dissemination and implementation of START-EDI guidelines. We will work with patient and public involvement representatives and under-served groups in research throughout the project stages.

**Ethics and dissemination:**

The study has received ethical approval from the Imperial College London Research Ethics Committee (study ID: 7592283). The reporting guidelines will be published in open access peer-reviewed publications and presented in international conferences, and disseminated through community networks and forums.

**Trial registration number:**

The project is pre-registered within the Open Science Framework (https://osf.io/8udbq/) and the Enhancing the Quality and Transparency of Health Research Network.

**STRENGTHS AND LIMITATIONS OF THIS STUDY**

- The development of equality, diversity and inclusion (EDI) reporting guidelines will follow the steps of the Enhancing the Quality and Transparency of Health Research Network toolkit.
- An international and multidisciplinary panel from diverse backgrounds will take part in the Delphi survey and consensus meeting.
- Although the Delphi process will be conducted in English, we will endeavour to recruit panellists from non-English-speaking regions, and the reporting guidelines will be made freely available in several languages.
- National context will be important to consider during this study, as access to data on EDI characteristics can be restricted by privacy laws and regulations in certain countries.
- The provision of EDI reporting guidelines does not necessarily translate to improved diversity and inclusivity in research. However, the reporting guidelines should raise awareness on EDI and encourage researchers to apply and report on EDI aspects throughout their entire research process.

## Introduction

 The importance of equality, diversity and inclusion (EDI) in health research has gained increasing recognition in recent years. Equality means ensuring every individual has an equal opportunity to make the most of their lives and talents, while diversity is the understanding that each individual is unique and these differences should be embraced. Inclusion is an effort and practice in which groups or individuals with different backgrounds are culturally and socially accepted in the community.^1^ Institutions and organisations, such as the National Health Service in England and the European Commission, have called for an improvement in diversity within research to ensure a greater understanding of effective treatments, devices and care for different societal groups.^2^,^4^

Certain minoritised groups are more likely to experience significant health disparities due to social and environmental factors, including age, sex, ethnicity, gender identity, geographical location, socio-economic status, sexual orientation, health literacy, language, education and insurance status.^5^,^9^ Data collected by the National Institute for Health and Care Research (NIHR) in the UK highlights that regions with the highest indices of deprivation and burden of disease have the lowest number of participants taking part in research.^10^ A review conducted by the NIHR of 148 randomised controlled trials (RCTs), published between 2019 and 2021, found that only one in seven (14%) patients enrolled in these trials were non-white.^11^ In the USA, the National Cancer Institute-Clinical Center data (2005–2020) also identified enrolment disparities with lower representation of older adults, women, Asian/Pacific Islander, American Indian/Alaska Native and Hispanic populations in cancer clinical trials.^12^

Barriers to inclusion of under-served and intersectional disadvantaged groups include narrow inclusion/exclusion eligibility criteria (eg, age, pregnancy), inaccessible promotion and communication materials (eg, providing written information in only one language), public lack of awareness of research, negative financial impacts or opportunity costs for participants, fear of not being treated with dignity and respect, and mistrust of the healthcare system.^213^,^15^ Exclusion of participants from certain backgrounds and inadequate reporting raises questions about applicability and generalisability of healthcare research findings to under-served groups.^16^ This may result in reduced access to new tests or therapies in these particular groups, leading to delayed diagnosis and suboptimal treatment.^17 18^ Bias may also arise due to inadequate representation of minoritised groups. It is essential to conduct studies that include diverse populations to investigate whether there is variability in treatment effects across subgroups. If, for example, the effect of a treatment is found to vary with age, sex or ethnicity, it is imperative that subsequent studies appropriately account for this in their design and reporting. This also acknowledges the potential for precision/personalised medicine where treatments may be tailored to individuals according to specific characteristics, such as sex or ethnicity.^19^

Reporting guidelines are regularly used in the literature, such as Standard Protocol Items: Recommendations for Interventional Trials (SPIRIT),^20^ Standards for Reporting of Diagnostic Accuracy (STARD),^21^ Transparent Reporting of a multivariable prediction model of Individual Prognosis Or Diagnosis-artificial intelligence (TRIPOD-AI),^22^ to improve the completeness and transparency of reporting. However, there are currently no universally accepted guidelines for the reporting of EDI considerations. While some EDI considerations are included in equity extensions of Consolidated Standards of Reporting Trials (CONSORT),^23^ Preferred Reporting Items for Systematic Reviews and Meta-Analyses (PRISMA)^24^ and Strengthening the Reporting of Observational Studies in Epidemiology (STROBE),^25^ these extensions are specifically designed for equity-focused studies or reviews and thus do not apply to all studies, leaving a gap in reporting guidance. Developing and implementing detailed EDI reporting guidelines across a broad range of study types is essential to ensure robust evaluation of the risk of bias (internal validity) and generalisability (external validity), as well as promoting fairness, transparency and reproducibility. This manuscript reports a protocol for the development of STAndards for ReporTing EDI (START-EDI), intended to provide a set of minimum items to help the research community describe EDI methods or techniques in studies with human participants. These guidelines will address the reporting of participant and population characteristics, and the reporting of any EDI-based interventions or processes.

## Methods and analysis

### Development of EDI reporting guidelines (START-EDI)

We plan to design the first EDI reporting guidelines which can be adopted across various research study types by several stakeholders, including researchers, authors, peer reviewers, journal editors and members of the public. These reporting guidelines are applicable to all research with direct involvement of human participants. The most common examples of relevant study types include diagnostic accuracy, clinical prediction models, epidemiological and efficacy/effectiveness studies (including RCTs) and study protocols.

**Figure 1:**
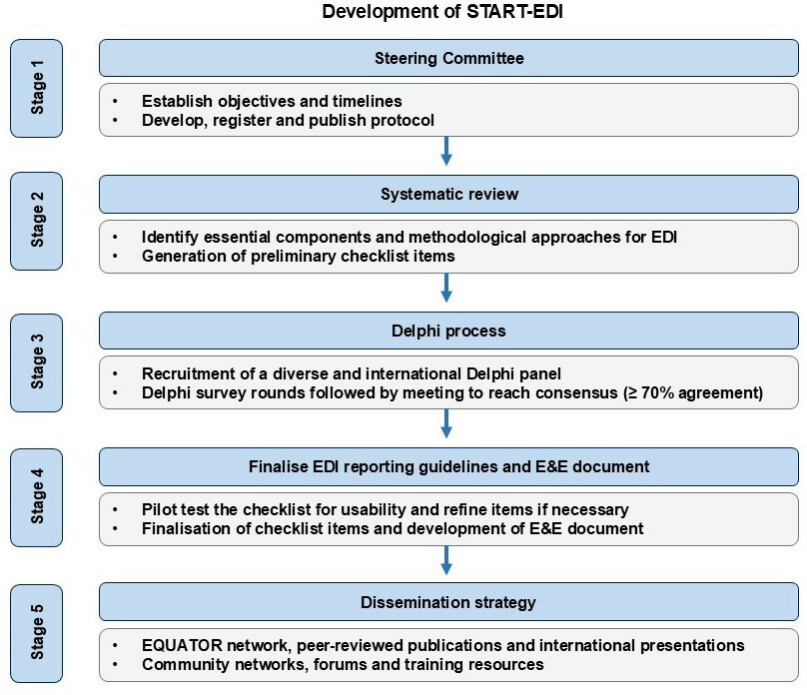
Overview of the development of Standards for Reporting Equality, Diversity and Inclusion (START-EDI) reporting guidelines. Abbreviations: E&E, explanation and elaboration document; EQUATOR, Enhancing the Quality and Transparency of Health Research.

The START-EDI project has been pre-registered within the Open Science Framework,^26^ and the guidelines will be developed in five key stages (figure 1). The study commenced in March 2025 and is due to complete by March 2027. The development of our guidelines will draw on steps provided in ‘Guidance for developers of health research reporting guidelines’^27^ and ACCORD (Accurate Consensus Reporting Document) guidelines.^28 29^ The study has been registered within the Enhancing the Quality and Transparency of Health Research (EQUATOR) Network,^30^ an international initiative that seeks to improve the reliability and value of medical research by promoting transparent and accurate reporting.

### Stage 1: Establishment of Steering Committee

The project will be overseen by an international multidisciplinary Steering Committee (MGF, HKL, PRB, RB, CN, AMM, PB, DAV, JH, PEJ, TR, BDN, AJC, LDS, SH, JFC, VW, PMMB and GBH) who have interest and expertise in EDI research. These committee members are a diverse group of primary and secondary care clinicians (MGF, PRB, DAV, BDN, JFC and GBH), methodologists (RB, CN, PB, SH and VW), qualitative researchers (HKL, RB, PB and TR), patient and public involvement and engagement (PPIE) leads (JH and PEJ), epidemiologists (AMM, AJC and JFC), statisticians (CN and LDS), reporting guideline developers (AMM, SH, JFC, VW and PMMB) and journal editorial staff (LDS, JFC, PMMB and GBH). The committee has links with centres and communities, beyond Europe and North America, in Africa, Asia, South America and Australia. The PPIE leads have co-designed the research proposal and methodology, as well as our public dissemination strategy. AMM has specifically led the development of the SPIRIT-Surrogate and CONSORT-Surrogate extensions,^31 32^ and SH led the development of the updated SPIRIT and CONSORT guidelines.^33^ JFC has expertise in the development of STARD,^21^ and VW led the development of CONSORT-Equity,^23^ PRISMA-Equity^24^ and STROBE-Equity^25^ guidelines. PMMB will play an important advisory role having vast experience and expertise in the development of reporting guideline initiatives such as STARD^21^ and PRISMA.^34^ A dedicated executive committee (MGF, HKL, PRB, JH, LDS, JFC, VW and GBH), a smaller group of Steering Committee members, will lead the guideline development process.

### Stage 2: Systematic review and generation of preliminary checklist items

A systematic review will be performed to identify and define the essential components of EDI in health research and methodological approaches or strategies for optimising diversity and inclusion. Eligible studies will therefore (i) describe or evaluate any intervention or initiative aimed at promoting EDI in research, (ii) describe how to overcome barriers to diversity or inclusion issues in the selection of research topics or participants or (iii) report on outcomes or impact of increased diversity and inclusion. We will also explore how EDI is defined across the literature, as a consensus-based definition of EDI will be required for the Delphi process and the final guidelines.

A literature search of MEDLINE, Embase, Emcare and CINAHL databases will be performed, and we will retrieve articles in the English language published over the last 5 years, due to an increasing awareness in EDI over this time period. Study designs such as RCTs, cohort, case-control, cross-sectional studies, qualitative studies and intervention development studies will be included. Social science outlets, grey literature, published policy or strategy documents on EDI over the last 5 years will also be searched. In addition, we will search for reporting guidelines, such as Reporting extension of CONSORT and SPIRIT for Inclusion, Diversity, Ethnicity and Race (RECONSIDER), which intends to focus on reporting of variables relevant to ethnically, culturally and linguistically diverse participants in RCTs.^35^

Two reviewers will independently screen the titles and abstracts retrieved, and any discrepancies will be resolved by a third reviewer. The following information will be extracted from the included studies where applicable: first author, year, country, study design, single or multicentre, definition of EDI, primary aims, target population, inclusion and exclusion criteria, details and outcomes of EDI intervention(s), encountered barriers and mitigating strategies, EDI characteristics reported, data collection and analysis strategy, dissemination and PPIE strategy. Information will be extracted using a data extraction form and grouped into themes for qualitative analysis. The review will therefore identify key themes on how EDI may be implemented in research and how it can affect outcomes. These findings will be summarised in tables and will be used to generate the preliminary list of checklist items for the Delphi process. We will also review prior relevant guidelines as sources for candidate items, eg, CONSORT-Equity^23^ and PRISMA-Equity^24^ and Sex and Gender Equity in Research (SAGER)^36^ guidelines.

### Stage 3: Delphi process

#### Study design

Reporting guidelines must be developed using an explicit methodology, which differentiates them from other efforts that produce a checklist, toolkit or roadmap.^27^ A consensus process is a crucial characteristic in the development of health research reporting guidelines. The Delphi methodology is a widely accepted technique for reaching a consensus and developing reporting guidelines.^37^,^39^ It enables the collation of international opinions while being cost-effective and reducing the need to travel. While a virtual Delphi approach provides participant anonymity that may allow for more open expression of views,^40^,^42^ it also has the potential disadvantage of a lack of group interaction for consensus building.^43^ Our final consensus meeting will therefore have an in-person element, as described below, to allow members to provide further clarification on some matters and present arguments to justify their viewpoints.

The potential checklist items generated by the systematic review will be agreed upon by the Steering Committee and will be put forward to the Delphi panel. A pilot by members of the committee will be conducted before the launch of the Delphi survey to improve the wording of items and logical flow and troubleshooting of any issues. Two Delphi survey rounds will be conducted, followed by the consensus meeting.^44^,^48^ However, if we fail to obtain consensus on most items after round two, we will consider a third survey round.^49^,^52^ The third round will only include items that have not reached a consensus by round two.

#### Sample size, recruitment and inclusion

**Figure 2:**
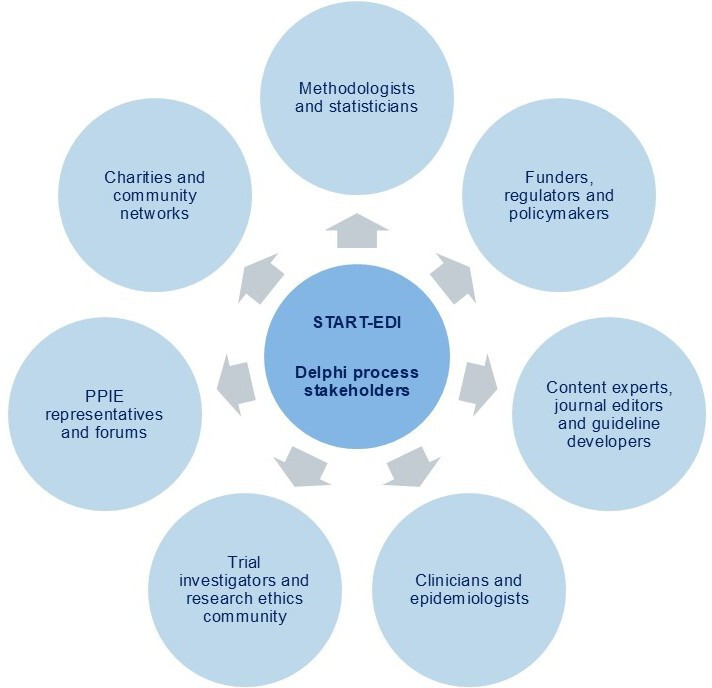
Key international stakeholder categories for Delphi process. Abbreviations: PPIE, patient and public involvement and engagement.

We will invite at least 100 international key stakeholders to take part in this Delphi process, including methodologists, statisticians, content experts, journal editors, epidemiologists, clinicians, trial investigators and coordinators, policymakers and patient and public representatives from diverse backgrounds, as well as members from the research ethics community, charities, community partners, research funding committees and the EQUATOR Network (figure 2). The START-EDI project will be advertised to potential Delphi panellists via networks, social media, conferences and meetings, relevant societies, organisations and journal editors. We will also invite corresponding authors of included articles from the systematic review and ask those participating to share the survey link with other people, networks or organisations that would be interested in participating. Based on observations from previous Delphi studies,^43 45 46 49^ we are targeting a minimum sample size of 60–80 to take part in the survey. This sample size has shown to have a high replicability of results to those of larger sample sizes.^52^ Ensuring diversity in backgrounds among Delphi panel members will be essential in the development of these universal reporting guidelines, not only from the academic and research community but also from charities and community networks. PPIE representatives and members of the public will be invited from community networks and charities (eg, Age UK, Reviving Links, Muslim Women Networks, South Asian Health Action and Amnesty International), patient and public engagement forums (eg, People in Research^53^ and the European Patients’ Forum^54^) and the MRC-NIHR Trials Methodology Research Partnership.^55^ The Steering Committee will endeavour to ensure a broad spread of representation across these stakeholder groups by collecting information on experience, demographics and geographical location and other EDI protected characteristics from the panellists, such as disability, gender reassignment, race and religion.^56^

#### Consensus definition

The guiding principles that will drive the selection of items and recommendations during the international Delphi process include inclusion, diversity, representation and external validity, internal validity and reproducibility. We will focus on items that should be reported in relevant study types, such as diagnostic accuracy, clinical prediction models and efficacy/effectiveness studies.

The first two rounds of the Delphi survey will be conducted online and facilitated by a blinded electronic voting platform (eg, Qualtrics XM software).^57^ Participants will be emailed a web link prompting them to complete the Delphi survey. The web link will access the survey with a short text section emphasising the importance of completing the exercise and a consent checkbox. Each round will be open for approximately 4 weeks, and an email reminder will be sent to participants to improve response rates if required.

For each item, the Delphi panel will provide their responses using a Likert scale. We will use the following Grading of Recommendations Assessment, Development and Evaluations (GRADE) 9-point scale^44^,^4649 58^:

- 1–3=of limited importance (item should not be included in the reporting guidelines).
- 4–6=important but not critical (item should be discussed).
- 7–9=important and critical (item should be included in the reporting guidelines).

We will also include a ‘don’t know’ option, for participants who do not feel qualified to rank any specific item. There will be two rounds of voting, where the responses will be analysed and summarised by the Steering Committee between each round. We will use the following consensus definitions^44 49^:

- Consensus for inclusion: ≥70% participants scoring 7–9 and <15% participants scoring 1–3.
- Consensus for exclusion: ≥70% participants scoring 1–3 and <15% participants scoring 7–9.
- No consensus for inclusion or exclusion: failure to achieve either of the above.

The Steering Committee will have the option to overrule the items that achieve a borderline level of consensus (eg, 65–69% scoring 7–9). Panellists will have the opportunity to suggest additional statements or modifications to the statements in free text fields. The committee will review items that do not achieve consensus in rounds one or two, and these will be revised or eliminated considering any free-text comments.

#### Consensus meeting

The aim of the hybrid consensus meeting will be to agree on the final items for inclusion in START-EDI reporting guidelines. The meeting will be held over 1-2 days, and participants will have the option to attend face-to-face or join virtually. Participants will be given the opportunity to register their interest in attending the consensus meeting at the end of the Delphi survey round two. The Steering Committee will select approximately 20 participants from those interested based on (i) their ability to attend the meeting on proposed dates and times; (ii) the requirement to have an international multidisciplinary group of stakeholders and (iii) the need to include members with a range of protected characteristics.^43 59^ Stakeholder group representatives (n=20) and Steering Committee members (n=19) will be invited to take part in the consensus meeting.

Prior to the consensus meeting, all participants will be sent the meeting agenda, participant list, summary of systematic review findings and the Delphi study results via email. The consensus meeting will (i) ratify inclusion of all items that reached consensus in the Delphi survey; (ii) discuss and vote for inclusion (≥70% voting to include or exclude) for items that did not reach consensus; (iii) discuss merging and wording of all items achieving inclusion to the final checklists. This round will be facilitated by the Steering Committee to provide structured interaction between individuals, enabling all members to have their voices heard. We will discuss and decide on the items to be included in the final guidelines and other agenda items such as developing a flow diagram, dissemination and implementation strategies, and future evaluation of developed outputs.

### Stage 4: Finalise START-EDI and explanation and elaboration (E&E) document

On completion of the Delphi process, the Steering Committee will finalise the START-EDI reporting guidelines and develop a flow diagram to enable authors to clearly and sequentially report information from their research study. The checklist will subsequently be pilot tested against a random selection of at least 40 peer-reviewed articles that had been identified from the systematic review to ensure usability and refine any checklist items if necessary.^60^ This will be carried out by experts in human factors and education, psychologists, language professionals and journal editorial staff. A separate E&E document will also be developed to provide a detailed rationale for the items included in the checklist, along with examples of good reporting.

### Stage 5: Dissemination strategy

The dissemination of START-EDI will be essential for implementation and encouraging adherence. The checklist and flow diagram will be freely available in an editable format and in several languages to promote diversity and inclusion. The final guidelines will be registered in the EQUATOR Network, and we will be in contact with editors of leading journals and funding organisations. We will also seek endorsement from groups of medical and surgical editors, such as the International Committee of Medical Journal Editors (ICMJE) and Surgery Journal Editors Group (SJEG). An online information package with worked examples will be circulated to international registered clinical trial units, Research and Development units, community networks and major charities that support clinical research. Lay summaries of START-EDI will also be published on websites (eg, patient groups, charities or governmental organisations) for the public.

START-EDI will be disseminated through open access peer-reviewed publications and presented at major international conferences, such as the Global Evidence Summit, International Clinical Trials Methodology Conference and the World Conference on Research Integrity and Peer Review. A dedicated group website will be created that contains webinars, training modules and additional resources explaining our guidelines for healthcare professionals and the public. We will engage with ethnic minority communities, religious leaders and collaborate with community partners to co-create and disseminate video resources in different languages explaining the importance of inclusivity in research and transparent reporting. We will also promote the guidelines through official social media channels (eg, LinkedIn and X), press releases and websites, such as the Campbell and Cochrane Equity Methods group. We plan to evaluate the impact of the reporting guidance through a systematic review (after 5 years) and measure web metrics to determine the number of associated citations; we will update the guidelines and provide extensions as necessary.

#### Patient and public involvement

PPIE will be embedded throughout all stages of the START-EDI project. Our PPIE strategy is led by JH and PEJ, who have co-developed the methodology for the development of the reporting guidelines. The results of the systematic review will be presented and discussed with the PPIE leads, and we will aim to identify 15–20 PPIE representatives from diverse backgrounds and under-served communities to take part in the Delphi process. Our PPIE leads will also help in the broad and inclusive dissemination of the guidelines. They have strong links to the National Institute for Health and Care Excellence Guideline Committee and the Diversity and Inclusion department at Sky Television and Media. We will formally assess the impact of PPIE involvement through the Guidance for Reporting Involvement of Patients and the Public (GRIPP2) checklist^61^ to reflect on how well we are meeting our aims and identify areas we can improve on.

#### Expression of interest

A short expression of interest form can be found here for those who are interested in participating in the Delphi survey rounds: https://forms.office.com/e/QxD8eLBDGH.

## Discussion

Recommendations have been suggested in the literature to promote inclusion to increase the enrolment of under-served populations in specific clinical trials, sometimes in the form of a toolkit or roadmap such as NIHR ‘Innovations in Clinical Trial Design and Delivery for the under-served’ (INCLUDE).^15 62^ These approaches, for example, include multilingual materials and staff, communication strategies and co-developing resources, staff cultural competency training, broad inclusion criteria, financial incentives, along with building community partnerships and increasing understanding and trust.^63 64^ On the other hand, the SAGER guidelines provide a thorough checklist for reporting on sex and gender information in research.^65^ START-EDI will provide guidance on how to report EDI in research in the abstract and introduction (eg, historical context), methods (eg, study design, recruitment, sites, PPIE and budgeting), results (eg, data analysis and presentation) and discussion (eg, interpretation of findings, dissemination and impact). The guidelines can therefore guide authors in designing their research and in preparing their manuscripts for submission, and they will also be useful for editors to assess EDI considerations in studies as part of the review process.

National context will be important to consider during this study, as access to data on EDI characteristics can be restricted by privacy laws and regulations in certain countries, as well as sensitivity associated with some identities, in particular ethnicity and sexual orientation. Countries may use different categories (eg, ethnicity categories) depending on their colonial, migration and race history. Having good international representation will be essential to making the guidelines as relevant as possible, for example, to parts of Asia and Africa where ethnicity, culture and research interests may be different. In addition, we will continue to take into account any geographical or political differences in the perception of EDI during the study itself, and we will provide any updated extensions in view of this in the future if necessary.

We anticipate that the START-EDI guidelines have the potential to lead to improved and consistent reporting of EDI. The guidelines will provide quality standards for EDI reporting for various clinical and epidemiological study types involving patients focusing on diagnostics and therapeutic interventions. We intend that these EDI reporting guidelines will ultimately be applicable to all biomedical and healthcare research.

### Ethics and dissemination

The study has received ethical approval from the Imperial College London Research Ethics Committee (study ID: 7592283). Informed consent and conflict of interest forms will be obtained from all participants in the Delphi process. START-EDI will be disseminated through conference presentations and open access peer-reviewed publications, along with community networks and charities as described in stage 5 (dissemination strategy).

### Conclusions

START-EDI will provide a list of minimum items that should be reported by healthcare researchers. It will improve understanding of equality and diversity within healthcare research and raise awareness of challenges to the inclusion of people from under-served and minoritised groups. We anticipate that START-EDI will benefit authors, editors, peer reviewers, funders, guideline developers, healthcare providers, policymakers, commissioners, patients and the public. It will guide researchers in considering EDI principles into their study design, data collection and interpretation, and the formation of research teams. The guidance will also lead to a more complete and transparent reporting of diversity by health researchers and provide a tool to assess the overall research design quality when reviewing materials submitted for funding and publication.
