# Supplementary material for: Development of START-EDI guidelines for reporting equality, diversity and inclusion in research: a study protocol
Source: BMJ Open. 2025 Jul 16;15(7):e095778. doi: 10.1136/bmjopen-2024-095778 (PMC12273130; doi:10.1136/bmjopen-2024-095778)
Supplement: Supplementary file 1 [file bmjopen-2024-095778.reviewer_comments.pdf]

PEER REVIEW HISTORY

BMJ Open publishes all reviews undertaken for accepted manuscripts. Reviewers are asked to complete a checklist review form and are provided with free text boxes to elaborate on their assessment. These free text comments are reproduced below.

ARTICLE DETAILS

Title (Provisional)

Development of START-EDI guidelines for reporting equality, diversity and inclusion in research: a study protocol

Authors

Fadel, Michael; Kettley-Linsell, Hannah; Boshier, Piers R; Barnes, Rebecca; Newby, Chris; Manyara, Anthony Muchai; Buckle, Peter; Vyas, Darshali A; Hepburn, Julie; Edgar-Jones, Philip; Rai, Tanvi; Nicholson, Brian D; Cross, Amanda J; Sharples, Linda; Hopewell, Sally; Cohen, Jérémie F.; Welch, Vivian A.; Bossuyt, Patrick MM; Hanna, George B

VERSION 1 - REVIEW

**Reviewer**	**1**
**Name**	**Lovell, Holly**
**Affiliation**	**Guy's and St Thomas' NHS Foundation Trust**
**Date**	**02-Apr-2025**
**COI**	**None**

This is a comprehensive and well written protocol for the development of novel guidance for the report of equality, diversity and inclusion (EDI) in research. The development of the proposed guidance is timely and important, given the current emphasis on developing research which incorporates EDI principles to ensure participants are representative to support generalisability.

The main point to address is that although the introduction provides a clear rationale for why EDI is important within research, it is not explicit what element of EDI in research the authors are focusing the EDI reporting guidelines on. It is started the focus is on the reporting of EDI methods or techniques, but further clarity is needed on what this encompasses. For example, is the focus of the proposed guidance on reporting of interventions or processes within research that aim to improve EDI? Or is the aim to provide guidance on reporting of participant and population characteristics to enable readers to better assess generalisability and EDI?

In addition there were two minor comments to address.

Study design - given it is an international sample, please state whether the Delphi survey will be available in different languages? If so, what processes will be in place to ensure accurate translation?

Sample size, recruitment and inclusion - It is stated patient and public representatives from diverse backgrounds will be invited. As EDI applies to a range of different characteristics, please include how diverse backgrounds will be defined or targeted? Will there be any monitoring or targets in place to ensure different characteristics are represented?

Otherwise this is a well thought out protocol with a clear methodology on an important and much needed topic.

Reviewer **2**

Name **Ross CBE, Fiona**

Affiliation **Kingston University and St. George's, University of London, Faculty of Health, Social Care and Education**

Date **12-May-2025**

COI **None**

I enjoyed reading this protocol. Some specific comments have been added to the text.

I thought the PPI was added somewhat abruptly at the end, I would have liked to have seen this centre stage and up front to demonstrate how the investigators intent to "embed" it.

The current weaponisation of EDI is some political jurisdictions has been developing at pace. The investigators need to think at an early stage how this is mitigated in order to ensure the study success. If it is not too late, maybe tailoring the language to inclusion rather than EDI.

VERSION 1 - AUTHOR RESPONSE

REVIEWER REPORT 1

Dr. Holly Lovell, Guy's and St Thomas' NHS Foundation Trust

This is a comprehensive and well written protocol for the development of novel guidance for the report of equality, diversity and inclusion (EDI) in research. The development of the proposed guidance is timely and important, given the current emphasis on developing research which incorporates EDI principles to ensure participants are representative to support generalisability.

Comment 1: The main point to address is that although the introduction provides a clear rationale for why EDI is important within research, it is not explicit what element of EDI in research the authors are focusing the EDI reporting guidelines on. It is started the focus is on the reporting of EDI methods or techniques, but further clarity is needed on what this encompasses. For example, is the focus of the proposed guidance on reporting of interventions or processes within research that aim to

improve EDI? Or is the aim to provide guidance on reporting of participant and population characteristics to enable readers to better assess generalisability and EDI?

Response: Thank you very much for your review and your comments.

We have further clarified that START-EDI intends to provide guidance on reporting of participant and population characteristics, as well as the reporting of any EDI based interventions or processes used [Introduction, page 6].

In addition there were two minor comments to address.

Comment 2: Study design - given it is an international sample, please state whether the Delphi survey will be available in different languages? If so, what processes will be in place to ensure accurate translation?

Response: The Delphi survey will be conducted in English. This has been mentioned as a potential limitation of the study, however, we will nevertheless endeavour to recruit panellists from non-English speaking regions [Strengths and limitation of this study, page 4].

Comment 3: Sample size, recruitment and inclusion - It is stated patient and public representatives from diverse backgrounds will be invited. As EDI applies to a range of different characteristics, please include how diverse backgrounds will be defined or targeted? Will there be any monitoring or targets in place to ensure different characteristics are represented?

Response: Thank you for your comments. Collected participant data from the first round of the Delphi process will be related to demographics, experience, and the protected characteristics defined under the Equality Act 2010, including age, disability, gender reassignment, race, religion or belief, sex, and sexual orientation [Methods, page 10]. We will endeavour to ensure that we recruit at least 2 people for each of these characteristics, with some intersectionality. The Steering Committee will also ensure we have representation from a diverse range of backgrounds and characteristics in the consensus meeting [Methods, page 12].

Otherwise this is a well thought out protocol with a clear methodology on an important and much needed topic.

REVIEWER REPORT 2

Prof. Fiona Ross CBE, Kingston University and St. George's, University of London

I enjoyed reading this protocol. Some specific comments have been added to the text.

Comment 1: I thought the PPI was added somewhat abruptly at the end, I would have liked to have seen this centre stage and up front to demonstrate how the investigators intent to "embed" it.

Response: Thank you for your review and your comments.

The “Patient and Public Involvement statement” was included in the Methods section as suggested by the journal authorship guidelines. We will be working with patient and public involvement and engagement (PPIE) representatives and under-served groups in research throughout all of the project stages [Abstract, page 3 and Methods, page 14].

We have two PPIE leads on the Steering Committee who have co-designed the protocol and this has now been made clearer in the manuscript [Methods, page 7]. We will be inviting PPIE representatives to take part in the Delphi process, who will also help in the dissemination of the reporting guidelines [Methods, page 9-10 and 13-14; Figure 2]. In addition, we will formally assess the impact of PPIE involvement and ensure our PPI practice is based on the best evidence.

Comment 2: The current weaponisation of EDI is some political jurisdictions has been developing at pace. The investigators need to think at an early stage how this is mitigated in order to ensure the study success. If it is not too late, maybe tailoring the language to inclusion rather than EDI.

Response: Thank you, we have considered your comments. We agree that national context will be very important to consider during the study, as access to data on EDI characteristics can be restricted by privacy laws and regulations in specific countries [Strengths and Limitations, page 4 and Discussion, page 15]. We feel that the terminology of equality, diversity and inclusion (EDI) helps to encompass our entire strategy. All of these components are essential to promoting good research practice and improving the relevance and quality of our research as described in the Introduction. Nevertheless, we will consider this and discuss terminology during the study itself as the field continues to progress,. This will also be reflected in the future with any updated guidance and extensions as necessary [Discussion, page 15].
